# Host Restriction of Lentiviruses and Viral Countermeasures: APOBEC3 and Vif

**DOI:** 10.3390/v5081934

**Published:** 2013-07-30

**Authors:** Stefán R. Jónsson, Valgerdur Andrésdóttir

**Affiliations:** Institute for Experimental Pathology, University of Iceland, Keldur, 112 Reykjavik, Iceland; E-Mail: stefanjo@hi.is

**Keywords:** small ruminant lentivirus, maedi-visna virus, APOBEC3, Vif, restriction factors, host defence

## Abstract

It is becoming increasingly clear that organisms have developed a variety of mechanisms to fight against viral infection. The viruses have developed means of counteracting these defences in various ways. The APOBEC3 proteins are a mammalian-specific family of nucleic acid cytidine deaminases that block retroviral infection. These inhibitors are counteracted by the Vif proteins encoded by most lentiviruses. In this paper, we will review the interaction of the lentiviral Vif proteins with the APOBEC3 proteins, with an emphasis on sheep APOBEC3 and maedi-visna virus (MVV) Vif.

## 1. Introduction

Lentiviruses encode a number of accessory proteins in addition to the Gag, Pol and Env proteins common to all retroviruses. Evidence is emerging that most of these accessory proteins function by inhibiting host restriction factors [1,2,3,4]. One of the accessory proteins is Vif, which is found in all lentiviruses, except equine infectious anemia viurs (EIAV). The *vif* gene encodes a small and highly basic protein rich in tryptophans [5,6]. Vif does not have a sequence resemblance to known proteins, and sequence similarity between the Vif proteins of lentiviruses is rather limited. Despite limited similarity, all Vif proteins have a conserved region in the C-terminal half, T/SLQXLA, which is necessary for their function [7]. Vif was implicated in a number of functions, but its precise role in viral infectivity remained elusive for a long time. 

## 2. The ΔVif Phenotype

HIV-1 Vif was initially shown to be essential for HIV-1 replication in human lymphocytes and a number of T-cell-derived cell lines termed non-permissive (e.g., CEM, H9 and Hut78), but it is not required for replication in so-called permissive cells, which include some human T-cell lines (SupT1, CEM-SS and Jurkat), as well as a number of non-haematopoietic cell lines, such as HeLa, COS7 and 293T [8,9,10]. It has also been shown that Vif is essential for replication in the natural target cells of HIV-2 [11], simian immunodeficiency virus (SIV) [12], feline immunodeficiency virus (FIV) [13,14], caprine arthritis encephalitis virus (CAEV) [15,16] and MVV [17].

The requirement for HIV-1 Vif is producer cell-dependent. Thus, ΔVif viruses from non-permissive cells are non-infectious, whether they are used to infect permissive or non-permissive cells, but ΔVif viruses produced in permissive cells are able to infect both permissive and non-permissive cell types [8,14,15,18,19,20,21,22]. 

Early on, two hypotheses were developed to explain the difference of importance of Vif in different cell types: one proposed the existence of an inhibitory factor in non-permissive cells; the other hypothesis proposed the existence of a positive factor in permissive cells that was able to perform the role of Vif. This was distinguished by fusing permissive and non-permissive cells. In these heterokaryons, the non-permissive phenotype was dominant, implying that the non-permissive cells contained a host factor that was a potent inhibitor of HIV-1, neutralized by Vif and absent in the permissive cell types [23,24]. This elusive host factor was later found to be human APOBEC3G, initially termed CEM15 [25], a member of a larger family of cytidine deaminases [26].

## 3. The APOBEC3 Family of Cytidine Deaminases

APOBEC3 (apolipoprotein B mRNA-editing catalytic polypeptide-like 3) proteins belong to a family of polynucleotide cytosine deaminases that includes AID (activation-induced deaminase), APOBEC1, APOBEC2, APOBEC3 and APOBEC4 [26,27,28]. APOBEC1 was the first family member to be identified, and therefore, the whole family gets its cumbersome name from its function. APOBEC1 is involved in lipid metabolism [29]. It is expressed in gastrointestinal tissues and specifically edits C6666 in apolipoprotein B mRNA to generate a premature stop codon that leads to a truncated functional form of apolipoprotein B [29,30]. 

The APOBEC3 (A3) proteins catalyse the deamination of cytosine to uracil in the minus-strand of viral DNA during reverse transcription, resulting in G-to-A mutation in the plus strand [31,32,33]. All family members have either one or two zinc-coordinating cytosine deaminase motifs (Z domains) comprising the sequence, His-Xaa-Glu-Xaa-_23–28_-Pro-Cys-Xaa_2–4_-Cys (where X can be nearly any amino acid), the histidine and two cysteine residues coordinate a Zn^2+^ ion and the glutamic acid residue is involved in proton shuttling. Catalysis results in hydrolytic deamination at the C4 position of the cytosine base, thereby converting cytosine to uracil [29,34]. Each Z domain belongs to one of three phylogenetic clusters, termed Z1, Z2 and Z3 [35,36].

**Figure 1:**
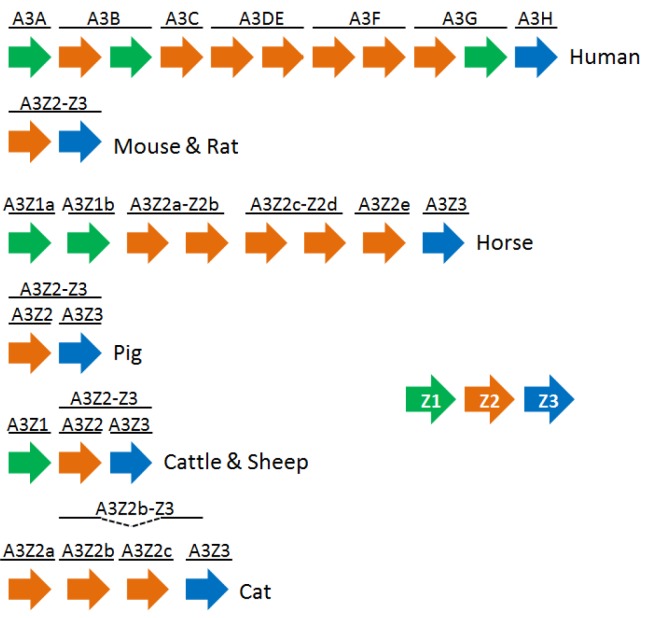
A schematic illustration of the A3 genes of the indicated mammals. Z1, Z2 and Z3 domains are shown in green, orange and blue, respectively. The illustration follows the colour scheme established by LaRue *et al.* [35,36].

The A3 genes are located between two conserved flanking genes, *CBX6* and *CBX7* [36]. The A3 genes vary in number among species, from one in rodents to seven in primates. Sheep and cattle have three A3 genes, which code for at least four active proteins [37]. The primate A3 proteins are named A3A through A3H, but a nomenclature system based on the Z-domains has been proposed for the non‑primate A3 genes [35,36] (Figure 1). Phylogenetic analysis suggests that the mammalian ancestor had a Z1-Z2-Z3 composition and that the primate A3 gene expansion occurred through a series of recombination events [36]. The pig, the mouse and the cat seem to have lost A3Z1 [35].

A3 proteins show a distinctive preference for deaminating cytosines in a specific sequence context. For instance, human A3G prefers the second C in CC dinucleotides (GG dinucleotides in the viral plus strand cDNA), whereas human A3F, A3H and A3B have a preference for the C of TC dinucleotides (GA in the viral cDNA) [31,37,38,39,40]. Mouse A3Z2-Z3 shows a preference for T-T/C-C [41]. Cow and sheep A3Z2-Z3 proteins have a trinucleotide preference for T-T/C-C [42] similar to that seen for the mouse A3Z2-Z3 protein [42]. 

It seems that although the mammalian A3 proteins have been under a strong positive selection and are highly flexible, they use a well conserved way of engaging retroelements, as A3 proteins have been shown to be active against elements in mammals, lower vertebrates and even in such distantly related organisms as yeast [43,44].

## 4. Inhibition of Viral Growth

In nonpermissive cells, A3G and A3F are packaged into budding virions and exert their activity in the target cells. During encapsidation, A3G interacts with the nucleocapsid (NC) of Gag, as well as binding to viral and/or small cellular RNA [45,46,47,48,49,50].

The mechanism of virus inhibition exerted by the A3 proteins has still not been fully elucidated. The massive hypermutation caused by A3G and A3F, as well as sheep A3Z2-Z3 [42] results in attenuation of the virus. However, many studies have shown that A3G and A3F can also inhibit infection without the aid of G to A hypermutation. This has been shown by observing that mutants with blocked editing activity, through mutation of any of the catalytically important residues in the C-terminal deamination domain, still inhibit HIV-1 infection to a significant extent [51]. Likewise, while the sheep and cow A3Z2-Z3 proteins had to have both deaminase domains intact for full levels of retrovirus restriction, a mutation in the active N-terminal deaminase domain retained some antiviral functions without obvious signs of retroviral hypermutation [42]. This deamination-independent restriction seems to be acting at reverse transcription and/or integration, but the underlying mechanism is unknown [52,53,54,55,56,57,58]. 

In the case of murine A3, restriction of the spread of murine leukaemia viruses and mouse mammary tumour virus seems to be exerted in the absence of hypermutation [59,60]. However, endogenous murine retroelements have been shown to contain G-A mutations in the target sequence characteristic of mA3 [41].

The relative contribution of the seven human A3 proteins to HIV restriction has been subject to some controversy. G to A mutations have been found in both 5'GG and 5'GA motifs in patient-derived HIV DNA sequences, implicating both A3G and any of the other human A3 proteins in virus restriction *in vivo*. Most studies have found that A3G is the main restriction factor that inhibits HIV-1 replication in primary target cells, followed by A3F and, possibly, A3DE [61,62]. There are at least seven haplotypes of A3H in humans, exhibiting diverse anti-viral activity against Vif-deficient HIV-1 [63]. A3H haplotype I is unstable and has no detectable antiviral activity, whereas A3H haplotype II, is active against HIV-1 [64]. The active haplotypes of A3H may have an antiviral role *in vivo*. On the other hand, A3A, A3B and A3C seem not to have a significant impact on HIV-1 replication [65]. A3A is preferentially expressed in cells of the myeloid lineage [66,67] and is not counteracted by HIV Vif. 

Recently, evidence has been accumulating to show that APOBEC3 proteins produced in the target cells can also exert inhibition. Thus, A3A potently restricts transfected DNA in primary monocytes [68,69], and also, A3A expressed in target cells has been shown to mediate G-A editing of HIV-1 cDNA [70]. Expression of A3A can lead to induction of DNA breaks and activation of the cellular DNA damage response (DDR), but it has been shown that triggering the DDR pathway acts as an alerting mechanism for the innate immune system [71,72]. Furthermore, expression of A3G in target cells has been shown to enhance the recognition of HIV-1 infected cells by NK cells through upregulation of NKG2D-activating ligands [73]. Thus, a picture is emerging, implicating the A3 proteins not only in retroviral restriction, but also in the broader innate immune response (further reviewed in [74]). 

It has been proposed that the A3 proteins can also have beneficial effect on the virus. The A3 proteins may contribute to the high mutation rate of the lentiviruses and, thus, facilitate escape from immune responses and antiviral drugs [75,76,77,78,79]. The relative contribution of reverse transcriptase and the A3 proteins to lentivirus variation is uncertain. Although A3G-mediated G-to-A mutations have been shown to have a detectable effect on HIV-1 evolution [78], it is likely that most of the variability of the lentiviruses is due to mistakes made by reverse transcriptase [80]. 

HIV DNA is heavily uracilated as a result of HIV reverse transcriptase not distinguishing between dUTP and dTTP. This has been shown to prevent the ends of the viral DNA integrating into the viral DNA itself instead of integrating into the chromosome (so-called autointegration) [81]. Uracilation of HIV DNA by A3G also inhibited autointegration [81]. However, the detrimental mutagenesis caused by the A3 proteins must outweigh the beneficial effects of the prevention of suicidal autointegration. 

## 5. Modes of Viral Escape

**Figure 2:**
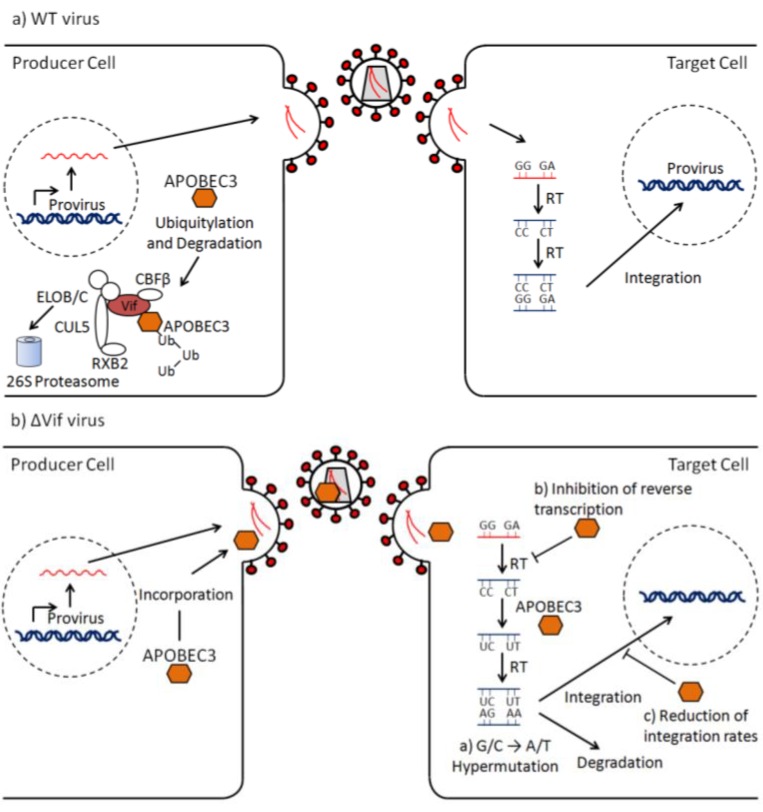
Schematic representation of the mechanisms of action of the HIV-1 Vif protein and APOBEC3. (**a**) Vif-induced degradation of APOBEC3 in infection by a WT virus (with a functional Vif protein). (**b**) APOBEC3 incorporation into ΔVif virions and antiviral effects in the target cell. See text for further details.

As mentioned earlier, all lentiviruses, except EIAV, code for Vif, which is a potent A3 antagonist. The main focus of research has been on the HIV-1 Vif-A3G interaction, which was the initial observation giving a clue to the role of Vif [25] (Figure 2). However, all human A3 proteins, except A3A and A3B, are sensitive to HIV-1 Vif [65,67]. Vif induces polyubiquitylation of A3G for subsequent degradation in the proteasome, thus preventing the incorporation of A3G into the viral particle [33,82,83,84]. Vif achieves this by acting as an adaptor connecting A3G and the cullin5-ElonginB-ElonginC-Rbx ubiquitin ligase [84]. The conserved T/SLQXLA motif in the C-terminus of Vif interacts with ElonginC and ElonginB and is referred to as the BC box [84,85]. A zinc-binding motif, Hx_5_Cx_17–18_Cx_3–5_H, which is conserved within the primate lentiviruses, together with a downstream LPx_4_L motif, mediates Cul5 interaction [86,87]. Recently, the cellular transcription factor core-binding factor-beta (CBFβ) was found to be required for HIV-1 Vif-mediated degradation of A3G [88,89]. HIV-1 Vif requires CBFβ to neutralize not only A3G, but the entire repertoire of Vif-sensitive human A3 proteins, and it is likely that CBFβ is an integral part of the Vif-E3 ligase assembly [90].

## 6. Ovine A3 Proteins and MVV Vif

Sheep code for at least four A3 proteins from three genes, A3Z1, A3Z2 and A3Z3, and a double domain protein, A3Z2-Z3 (Figure 1). All four proteins elicit DNA cytosine deaminase activity [36]. The human A3 proteins that have shown the most potent activity against HIV-1, A3G and A3F have two deaminase domains. The mouse A3 similarly has two deaminase domains. The sheep A3Z2-Z3 double domain protein restricts HIV-1 and murine leukemia virus (MLV), as well as a Vif-deleted MVV. However, unlike human A3G and A3F, which have a C-terminal active cytosine deaminase domain, the sheep and cattle A3Z2-Z3 proteins have an active N-terminal deaminase domain [42]. 

The human A3 proteins each have characteristic subcellular distributions, likely correlating with their different functions. Similarly, the subcellular distribution of the sheep A3 proteins may give a clue to their function. The sheep A3Z2-Z3 protein, as well as the A3Z-Z3 proteins of cattle and pigs have a predominantly cytoplasmic distribution. This subcellular distribution correlates with that of human A3F and A3G and may reflect a similar function [36,42]. Human A3A, on the other hand, has a cell-wide localization with a nuclear bias, and the cow A3Z1 localizes very similarly. Likewise, the A3Z3 proteins of cattle and sheep localize mostly in the cytoplasm, like the human A3H HapII [36,91]. It can therefore be anticipated that sheep A3Z1 corresponds to A3A, sheep A3Z3 corresponds to A3H and sheep A3Z2-Z3 corresponds to A3F and A3G. 

MVV Vif is essential for MVV replication in sheep blood-derived macrophages and *in vivo* [17] and has been shown to neutralize sheep A3Z2-Z3, as well as sheep A3Z3 [92,93]. Whereas the A3 proteins restrict a broad range of retroviruses and retroviral elements and the restriction is not species‑specific, the Vif proteins usually counteract the restriction activity of the A3 proteins of their host species. Thus, sheep A3Z2-Z3 restricts HIV-1, as well as Vif-deficient MVV, but MVV Vif can only degrade sheep A3Z2-Z3, but not A3G [42]. However, the A3H/A3Z3 proteins present a special case; here, the Vif protein of MVV seems to be especially promiscuous, degrading not only sheep A3Z3, but also the A3Z3-type proteins of humans, macaques, cows and cats. This is different from the closely related BIV Vif, as well as the Vif proteins of HIV-1 and FIV that appear strictly specific to the A3Z3-type proteins of their hosts. SIVmac Vif is intermediate, mediating the degradation of the human and cow A3Z3-type proteins in addition to the macaque one. The mechanism behind this difference in specificity remains to be elucidated [92]. 

All Vif proteins have a BC box in common. In MVV Vif, this sequence is SLQRLA. Mutating the SLQ to AAA results in the inability of MVV Vif to degrade sheep A3, indicating that MVV Vif uses a similar mechanism as the primate Vif proteins to degrade A3. However, MVV Vif lacks the HCCH motif found in primate Vifs and also a consensus Cul5 box [94], and there is evidence to suggest that MVV Vif binds more strongly to Cul2 than to Cul5 [95]. There is a sequence downstream from the BC box similar to a consensus Cul2 box, but we have found that mutating this sequence did not abolish Vif-mediated degradation of sheep A3Z2-Z3 [96]. It is, therefore, most likely that the MVV Vif protein presents a novel way of binding a ubiquitin ligase complex. Similarly, FIV has been shown to use a Cul5 ubiquitin ligase complex to degrade feline A3 in the absence of a consensus Cul5 box [97].

We have obtained evidence to suggest that neutralizing A3 is not the sole function of MVV Vif. A pro-ser mutation in the C-terminus of Vif together with a leu to arg substitution in CA attenuates viral replication in blood-derived macrophages and *in vivo* without inducing hypermutation. These mutations may define a novel restriction factor that targets the CA and is counteracted by Vif [98]. Early studies of the HIV-1ΔVif phenotype in non-permissive cells revealed morphological defects, as well as the instability of the viral core [99,100,101]. This may be a consequence of A3 expression, but it could also be due to a restriction factor that has not been identified yet.

## 7. Concluding Remarks

Antiretroviral factors are active against a broad range of retroviral elements, suggesting that defence mechanisms found in one system can be applied to other systems. Studies on host restriction factors of the small ruminants and the Vif proteins of the SLRVs can, thus, advance our understanding of the human restriction factors and HIV.

Although the human APOBEC3 proteins have been much studied, there are still a number of open questions about their mechanisms of action. These include the mechanism of deamination-independent inhibition, which both A3G and sheep A3Z2-Z3 exhibit; and the mechanism of A3A action, which may have its counterpart in sheep A3Z1, both in restricting foreign DNA and in inhibiting virus replication in myeloid target cells.

Comparative studies on the Vif proteins of the various primate and non-primate lentiviruses will bring to light similarities, as well as dissimilarities that may be informative. These proteins seem to have evolved different ways of using the ubiquitin ligase complexes for degrading the A3 proteins. Studies on the Vif proteins may unravel novel functions in addition to their prominent roles in counteracting cellular A3 proteins.
